# Inquiries into the Healthy and Diseased Appearances of the Mucous Membrane of the Stomach and Intestines

**Published:** 1828-03

**Authors:** W. E. Horner

**Affiliations:** adjunct Professor of Anatomy in the University of Pennsylvania.


### STOMACH AND INTESTINES.

*Inquiries into the Healthy and Diseased Appearances of the Mucous Membrane of the Stomach and Intestines.* By W. E. Horner, M.D. adjunct Professor of Anatomy in the University of Pennsylvania. (Condensed from the *American Journal of the Medical Sciences.*)

(Continued from page 121.)

### III. *Of the Red Inflammation of Mucous Membranes.*

Red inflammation has for its symptoms heat, pain, redness, and swelling, all of which are very obvious when it attacks a portion of the external surface of the body. It differs from congestion, in the latter causing an accumulation of blood in all the capillaries of an organ in which the blood is arrested, while inflammation is most frequently limited to a single tissue, and exhibits redness in it alone, or at least principally; as, for example, in mucous coats during their inflammation, in muscular during theirs, in peritoneal during theirs, &c. This, however, must depend upon the intensity of the inflammation, for, where it is very considerable, adjacent coats will also be affected.

Congestion is regular in its progress, and begins without constitutional symptoms; whereas inflammation begins with a chill, has exacerbations and remissions, and a course of augmentation, of stasis, and of decline. They both may terminate by delitescence, or a disappearance of the accumulated blood, or by resolution when particles of red blood are infiltrated; but inflammation also terminates in suppuration, in scirrhus, in hepatization, and softening of a part.

The traces of acute inflammation are in many cases very fugitive, and entirely disappear upon death, because the local irritation, which attracted the blood and accumulated it, having ceased, the blood abandons that part and retires towards the centre of the circulation. We can seldom tell by the appearances twenty-four hours after death, the quantity of blood which has penetrated an inflamed membrane, as the peritoneum, the pleura, the cellular and mucous membranes, the skin, &c. The eruption of measles, and the redness of sorethroat, disappear on the death of the patient. We are not, however, to infer from these circumstances that the mere afflux of blood to a part constitutes inflammation: on the contrary, it is attended with a dilated condition of the vessels independent of this afflux, for, if death occur during the height of measles, the eruptions may be made to reappear by injecting the vessels.

There are many proofs of inflammation being seated in the capillary system, and of the circulation of the latter being in some measure independent of the general circulation. 1. The partial erythemas of the skin. 2. In slight incisions or scratches, the skin will bleed very little at first, but, so soon as the irritation has determined a flow of blood to the part, the hemorrhage is sometimes troublesome, as in shaving. 3. The secondary bleeding from operations is sometimes very profuse from this cause. 4. Another, and a very strong proof of the power of the capillary circulation to go on without the impulsion from the heart, is that, if a limb be made turgid with blood by the application of a tourniquet, as in common amputation, when it is cut off, though it be completely withdrawn from the influence of the heart, yet the capillaries empty themselves, and the limb becomes as pallid as under any other circumstances of death. Though I feel satisfied of the capillary circulation being different from the circulation in larger vessels in not depending wholly on the heart, yet it is to be understood that I do not withdraw it entirely from this agency, for there are many strong proofs of its subordinancy. In the inflammation of transparent parts, for example, of the skin under the finger-nail, where vascularity is very perceptible, every pulsation of the arteries of the upper extremity is attended for the moment by an increase in the vascularity of the part inflamed, and a deeper suffusion of it.

The irregularities in the capillary circulation are always partial, for if the blood move more slowly or go backwards in one place, it will proceed more speedily in another, so as to maintain the general circulation: if it were otherwise, life must cease, for it would be incompatible with it for all the capillary circulation to be arrested or to go backwards at once. Hence arises a law of organism always strongly exemplified in disease, that, if the vital forces augment their energy at one place, they diminish it in another. There appears, indeed, to be only a certain quantity allotted to each individual, and though it may be accumulated at certain points so as to present a disproportionate sum in different parts of the body, yet it never can be augmented or diminished in mass by disease. This principle is the foundation of all correct physiology and pathology, and is in fact a matter of daily observation. We learn from this why it is that general bleeding is frequently inefficient in local inflammation; for so long as local irritation continues, whereby blood is attracted (not driven) to a part, the mass of blood may be diminished one quarter, and yet the inflammation will continue: whereas, in an animal having no irritated point in his system, you may double the quantity of blood by transfusion, and yet not produce local inflammation.*

Every operation of nature manifests an economical or exact application of means to effect an object: there is nothing superfluous. We therefore see that, during the waking state, the vital energies and the currents of blood are principally directed towards the senses and organs of animal life or those of relation, as the muscles, &c.; and that, during sleep, the vital energies are applied to molecular nutrition, and the repairs and general restoration of equilibrium in the system. Infants sleep more than adults, for the reason that their molecular nutrition or growth is more imperiously required. Deprive any one of sleep, and he soon becomes haggard and emaciated. There is a continued alternation in each individual in the actions of the system and in the currents of blood, depending upon the waking and upon the sleeping states,—one set of organs requiring the blood more at one time, and another set at another. It is impossible for an undue excitation and an undue rush of blood to occur upon all points of the body at one and the same time, or, in other words, for there to be a general disease. A general disease or inflammation would be as little consistent with nature, as a general rise in the waters of the ocean, which we know to be always lowered at one place when they are raised by the wind or other causes in another. Such an inference would be a solecism in pathology, for the reason that disease implies a loss of equilibrium in the actions of the body, by one or more parts having an unnatural preponderance. The general actions of life may all be increased at the same time, as one increases the weights upon each end of a pair of scales, and yet preserves an equilibrium; but, so soon as we take from one or add to the other, the equality is destroyed.

It is, perhaps, owing to molecular nutrition being stronger at night, that the symptoms of inflammation (which at least in its milder forms bears some analogy) are increased at this time.

The general augmentation or acceleration of the actions of the body in exercise, as running, jumping, and so on, differs from disease in this respect, that there is no concentration of irritability in any one organ, and a consequent afflux of blood; but, on the contrary, the equilibrium of the system is maintained, notwithstanding the apparent haste and momentum of its actions.

The causes which produce inflammation of the mucous

* Anat. Gen. vol. ii. p. 41

coat of the stomach and bowels are numerous, and in many individuals there is an idiosyncrasy which inclines them to it, from very slight causes. Persons who are exposed to the changes of the atmosphere by sleeping out of doors, as in military life, present frequent examples of it: bad aliment, or an excessive indulgence in what is good, also disposes to it. Poisons of all kinds may produce it; also moral affections, as melancholy, rage, &c. As my principal object, however, is to point out the anatomical characters which are left after death by gastro-enteritis, I must confine myself to such remarks as are pertinent to them.

In acute inflammation of the mucous membrane of the stomach, when the patient dies in the early stage of the disease, the blood-vessels which ramify through the stomach are enlarged and distended with blood. The membrane itself is covered by a coat of mucus, which is sometimes limpid like the white of an egg, but on other occasions thick and purulent like that from the nose. The mucus adheres frequently very strongly to the stomach, and is now and then so tenacious and consistent, from the admixture of coagulating lymph with it, as to resemble a false membrane. When the coat of mucus is scraped and washed away, the mucous membrane itself is brought into view, being most frequently, in the greater part of its extent, of a deep red, approaching on some occasions a crimson red, on others a purple or black. These colours are owing to the injection of a prodigious number of capillary vessels in the mucous coat; and, in addition to them, we find the inflamed part of the stomach interspersed with bands and patches of red, of the colour of coagulated blood, being a species of ecchymosis, in which the blood has escaped beneath, and in the substance of the mucous membrane. The mucous membrane at these places is somewhat softer than natural, and sometimes appears swollen, may be readily detached from the cellular coat with the end of a scalpel, and is in the condition of a *bouillie.* In cutting through all the coats of the stomach, and looking at the incised edge, it will be seen that, where the general and diffused redness exists, the colour is only superficial; but, at the ecchymosed spots, not only the whole thickness of the mucous coat is concerned, but even the corresponding part of the muscular, is more highly coloured than in common. In some cases where the gastritis has been occasioned by caustics, there are eschars of the mucous membrane, some of which are detached and leave the muscular, or even the peritoneal coat bare, from the depth of the impression made. It is said that such eschars become more distinct some hours after the stomach has been exposed to the air.*

In some cases of acute inflammation, where the symptoms have been those of army dysentery or of typhous fever, the stomach and bowels are occasionally found a little thicker than usual, and of a yellowish brown or red colour on their peritoneal surface, and in their thickness; the fetor from them, on opening the abdomen, is much greater than usual, and the peculiar smell of the halitus from the peritoneum is overcome by it.

Some circumstances have been alluded to by Drs. Physick and Cathrall,† which, if properly appreciated, would contribute much to settle the opinions of medical men in relation to gastric inflammation; for they go to show that the appearance of the inflammation of yellow fever varies according to the date of the disease. "In two persons who died of this disease on the fifth day, the villous membrane of the stomach, especially about its smaller end, was found highly inflamed, and this inflammation extended through the pylorus into the duodenum some way." The inflammation was exactly similar to that produced by arsenic.

"In another person, who died on the eighth day of the disease, several spots of extravasation were discovered between the membranes, particularly about the smaller end of the stomach, the inflammation of which had considerably abated. Pus was seen in the beginning of the duodenum, and the villous membrane at this part was thickened."

"In two other persons who died at a more advanced period of the disease, the stomach appeared spotted in many places with extravasations, and the inflammation disappeared." It and the intestines contained a black liquor so acrid as to produce inflammation and swelling on the operator's hands, which continued for some days.

"The stomach of those who died early in this disease was always contracted; but, in those who died at a more advanced period, where extravasations appeared, it was distended with air." The external surface of the stomach was healthy, but, from its veins being distended with blood, they had a dark appearance.

In the dissections performed by Dr. Physick in the years 1798-99,‡ the inside of the stomach in some cases resembled the black vomit precisely in colour. In most of these cases,

* Dict. des Sc. Med. t. xvii.

† Rush's Inquiries, vol. iii. p. 172. Philadelphia, 1809.

‡ Medical Repository, New York, vol. v. p. 129.

no black matter was found in the cavity of the stomach, but the blackness depended upon this fluid being retained in the vessels of the inflamed mucous membrane. The Doctor remarks, that he never observed any putridity attending it, and that the colour was very distinguishable from the dark purple of gangrene. In some stomachs, the blackness was universal; in others, in spots only, there being some spots in a state of high inflammation, and giving the inside of the stomach a chequered appearance. These spots, in one instance, resembled one another precisely in shape, and in all other respects, excepting colour, in which they differed, one being black and the other red.

Dr. Physick's opinion on these subjects was, that this black matter, commonly called the black vomit from its being ejected by vomiting, was a secretion from the inflamed vessels of the stomach, and one of the most common modes by which violent inflammation of the stomach has a disposition to terminate. "For in some cases where the vomiting of black matter had been considerable in quantity, or continued for several days, the inflammation was found very faint indeed; and in some the inside of the stomach appeared as if covered over with a vast number of small glands, like mucous follicles crowded together."*

When chronic inflammation has assailed the mucous coat of the stomach, this membrane is commonly found thrown into numerous folds. Sometimes it is thickened, of a denser texture than natural, and reddish, with irregular white patches. On other occasions, the whole of it is red, with purple spots, as in acute gastritis; sometimes the whole of it is of a purple, approaching to claret colour. When the disease has been produced by poisons of a force insufficient to cause immediate death, small ulcers are found near the pylorus, and along the greater curvature of the stomach.†

In some observations that I have made on the stomachs of intemperate persons at the Alms-house, I have found the mucous coat thickened and dense, without any remarkable contraction of the stomach, yet thrown into numerous, thick, elevated rugæ, and the summits of those rugæ so reddened by numerous capillary vessels injected with blood, that, at the distance of a few feet, they appeared, when the distinction of the individual capillaries was lost in the distance, like red streaks. This, which is probably one of the pathognomonic signs of a recent debauch in an old drunkard, is frequently mis-

* Medical Repository, New York, vol. v. p. 129.

† Dict. des Sc. Med. tom. xvii. p. 379.

taken, in the post-mortem examinations at the Alms-house, for congestion, from an arrest of blood in the agonies of dissolution.

The red blotches which form the leading anatomical character of acute mucous inflammation in malignant fevers, may be readily produced by chemical irritants.

*Experiment 6th.*—A full-grown white rabbit, fed three hours before on cabbage-leaf, was fixed in a frame, and the abdomen opened by a cut from the sternum to the pubes. The stomach was drawn out: it was filled with the food, which exhibited the appearance of having been boiled, and had an hour-glass contraction in its middle, separating it into two sacs, with a small orifice between them. The stomach was opened all along its greater curvature, and the food turned out: the whole mucous coat was of a scarlet-lake colour. This colour became deep crimson-lake, and the stomach more suffused with blood on wiping or rubbing the mucous coat with the hand. To one part I applied the glass stopper of a bottle, wet with a saturated solution of corrosive sublimate in Sp. Vin. rect., which had the effect, in three or four minutes, of deepening the colour of that part still more.

The colon was in greater part filled with soft fæces. The small intestines, on being cut into, exhibited the mucous coat of a light-lake, approaching vermilion, which colour remained after death.

In this experiment, an ounce and a half of blood was lost from the cut vessels. I had proceeded to open the trachea, and had fixed a pipe into it for the purpose of trying the effect upon the blood of shutting off the air from the lungs, when the animal died after a faint struggle.

In five minutes after, the stomach was cut out, and put under a stream of water to wash it well. The lake colour still remained in the whole mucous coat, from the residence of red blood in the capillary system. At the place where the corrosive sublimate was applied, there were irregular, oblong, red blotches of two or three lines broad. The mucous coat had, indeed, this variety of hue in other places also, but not so deep. In one part there was a line of petechial spots, half an inch or three-quarters in length, from half a line to a line in diameter, each resembling in colour the red blotches.

*Observation 4th.—Acute Gastritis and Peritonitis.*—Harriet Derrickson, an African, aged twenty-six years, was received near the term of pregnancy into the Alms-house, July 13th, 1827, after a recent confinement in prison. On the preceding day she had got wet, which produced a pain in the right hypochondrium: she was relieved by immediate bleeding.

July 13th.—Her pulse was rapid and febrile, and skin rather above natural heat, but the symptoms were generally mild, and not alarming. She was ordered a dose of Epsom salts, and barley water for common drink.

14th.—Eye slightly sallow; tongue inclined to dryness, with dark fur at base; costiveness; skin of common temperature, and moist; pulse rather tense and rapid. In the afternoon, there was a violent pain over the whole head, which was rendered still more severe upon her stooping. Vertigo, thirst, loss of appetite, pains in the limbs. A dose of Seidlitz powders, and cold applications to her head were prescribed.

15th.—Tongue unchanged; thirsty still; skin hot; violent pain in the right side; eyes yellow; breathing hurried; pulse rapid, not strong. Treatment continued, with the addition of cups to the right hypochondrium.

16th.—She miscarried of a dead child today at six A.M. with the loss of but very little blood, but her strength was almost exhausted. Her skin continued hot and dry, her tongue dry; stupidity. The symptoms continued to increase during the day.—Twenty leeches were applied to the temples, and forty to the epigastrium.

At four P.M. a saline mixture, which she had taken in the morning, brought away some natural evacuations; but the skin continued excessively hot; the pulse rapid, without tension, and compressible; the tongue still furred with red apex and edges. The sense of pain in the head had diminished much, but there was great tenderness of the epigastric and hypochondriac regions.—A blister was applied to her side, sinapisms to her ankles, and fifteen drops of spirit of turpentine were directed every hour.

17th.—Almost entire insensibility; eyes of a golden colour; pulse very feeble; soreness of flesh; restlessness and moaning.—Spirit of turpentine increased in the afternoon to ℨij. every hour.—She died at six and a half P.M.

This case was not treated by myself: the notes of it, so far, were communicated by a most zealous and intelligent student of the house, Dr. ASHMEAD.

*Autopsy*, eighteen hours after death, by Drs. Hodge, Ashmead, and myself.—We found the internal face of the uterus lined with red blood, which had blended with its ragged surface; clots of blood were in the uterine orifices of the uterine veins, and could be readily picked out. There had been no undue hemorrhage or lochial discharge.

The capillaries of the peritoneum, especially at the lower part of the abdomen, were distended with blood, and produced here and there rose-coloured patches. The peritoneal covering of the uterus seemed to have exceeded other parts in the intenseness of its disease, and had beneath it several patches of from six to twelve lines each of ecchymosed blood.

The stomach presented a degree of inflammation over its whole mucous coat, not often surpassed even in yellow fever. Some inches square of its middle were almost in a state of sphacelation, from the congestion and extravasation of red blood. Small clots of black blood were found among the contents of the stomach, but there was no black vomit.

The following case will serve to illustrate the appearance of the mucous membrane of the stomach, after an inflammation of two or three weeks.

*Observation 5th.—Chronic Gastritis.*—Women's Surgical Ward, Alms-house. Mary M'Graw, aged twenty-eight, moderately corpulent, common stature, lymphatic temperament, habits of intemperance, was admitted May 10th, 1827, for a fracture of the neck of the acromion scapulæ, destruction of the acromio-clavicular articulation, and an extensive laceration and injury to the shoulder joint, manifested by the extreme facility with which it could be thrown out of place and restored again. The account she gave was, that two years ago she fell into a ditch, upon which occasion she produced the injury mentioned, and also broke the os humeri about the lower end of the bicipital groove. The os humeri was cured of its fracture by the attention of a surgeon, but the shoulder remained in the state described. She afterwards continued to do her ordinary work till last January, when she began to suffer violent pain in the affected shoulder, and subsequently lost the use of her arm.

Without very sanguine hopes of the success of the treatment, I yet determined, as there was not much probability of the individual being made more helpless by the operation, to try the effects of a seton in producing a reunion of the broken acromion and of the acromio-clavicular articulation. The patient agreed to the proposal, and May 29th was fixed for its execution, but, owing to her being feverish and unwell on that day, it was postponed to the next prescribing day.

June 1st.—The patient seeming sufficiently well, I passed a seton through the acromio-clavicular articulation, and another through the fractured neck of the acromion. The patient, being exposed in returning through the yard to her ward, was seized directly afterwards with a chill, which lasted half an hour, and was succeeded by fever. (Of which she died, on the 21st.)

*Autopsy.*—Abdomen: The peritoneal surface of this cavity and of its viscera, universally of a clear white pearl colour, and destitute of adhesions.

Stomach: Moderately distended with flatus, and contained the last articles of medicine and of nourishment. Its mucous coat, for the most part, of a white colour, and destitute of injected vessels; yet in the cardiac half, particularly the cul de sac, many of the large veins were occupied with blood, and led to blotches of blood now almost removed, but which were yet sufficiently obvious when the part was held between the eye and a window; mucous coat in left half so softened that it could be readily scraped away with the finger.

Intestines healthy, light-coloured, and diaphanous, excepting the duodenum, the internal coat of which was much injected, hardened, thickened, and with much less appearance of valvulæ conniventes than usual.

Liver of a brownish yellow colour, as in intemperate persons, somewhat indurated, and had on its upper surface a hydatid the size of a nutmeg.

This case, in which there was unequivocally marked gastric irritation, proves that when an acute inflammation of the stomach had persisted for some days, though it terminates in death, no very great redness of the internal membrane may be manifest, and consequently that it is impossible to estimate the state of irritation of an organ during life solely by the quantity of blood left in it after death. Dying seems to have the effect of concentrating more and more towards the heart the vital powers and the fluids, or, in other words, withdrawing them from the circumference to the centre; in the same way that a prudent general, on finding his outposts too much extended for the size of his army, will contract them more and more, as his force diminishes by battle and disease.

[To be continued.]

